# The Effect of Smartphone Use and Nomophobia on Sleep Quality and Daytime Sleepiness in Turkey

**DOI:** 10.1093/eurpub/ckac131.242

**Published:** 2022-10-25

**Authors:** B Erten, E Pehlivan, E Yalcin

**Affiliations:** 1 Public Health, Balikesir Provincial Health Department, Altieylul, Balikesir, Turkey; 2 School of Medicine, Inonu University, Battalgazi, Malatya, Turkey

## Abstract

**Background:**

Smartphones used unconsciously and in an uncontrolled manner make young people experience sleep problems. This study aimed to investigate the effects of university students’ smartphone addiction and nomophobia levels on sleep quality and excessive daytime sleepiness.

**Methods:**

This study, which had a cross-sectional design, was conducted with 390 people who were first-year and senior students at Inonu University between November and December 2019. The Pittsburgh Sleep Quality Index, Epworth Sleepiness Scale, Nomophobia Scale, and Smartphone Addiction Scale were used in the present study used. For statistical analysis, the chi-squared test, the Student’s t-test, one-way ANOVA, Spearman’s rank correlation coefficient and binomial logistic regression analysis were used. The research has ethics committee approval.The error level was chosen as p = 0.05.

**Results:**

The smartphone use time of the students was finded to be 5.4±2.6 years, daily online time was 4.3±2.6 hours, and daily sleep time was 7.4±1.5 hours. The students received 78.3±25.8 points from the Nomophobia Scale, 90.3±29.7 from the Smartphone Addiction Scale, 7.2±2.8 from the Pittsburgh Sleep Quality Index, and 5.9±4.1 points from the Epworth Sleepiness Scale. A total of 54.4% of students had moderate, and 22.8% had severe nomophobic symptoms; 83.6% of the group had poor sleep quality, and 14.6% had excessive daytime sleepiness. A positive, moderate and significant relation was detected between the mean Nomophobia score and the mean Smartphone Addiction Scale score. It was also determined that those with less than 30 minutes of smartphone use before sleeping had low nomophobia, smartphone addiction and daytime sleepiness scores, and better sleep quality.

**Conclusions:**

Nomophobic symptoms and smartphone addiction were observed to be high in university students. Most students had poor sleep quality. The awareness of students on healthy sleep, and conscious and controlled smartphone use should be increased.

**Key messages:**

• Smartphone addiction increases the level of nomophobia moderately in university students.

• Those who use a smartphone less than 30 minutes before going to sleep have better sleep quality and lower daytime sleepiness.

